# Analysis of Antimicrobial-Triggered Membrane Depolarization Using Voltage Sensitive Dyes

**DOI:** 10.3389/fcell.2016.00029

**Published:** 2016-04-13

**Authors:** J. Derk te Winkel, Declan A. Gray, Kenneth H. Seistrup, Leendert W. Hamoen, Henrik Strahl

**Affiliations:** ^1^Centre for Bacterial Cell Biology, Institute for Cell and Molecular Biosciences, Newcastle UniversityNewcastle upon Tyne, UK; ^2^Swammerdam Institute for Life Sciences, University of AmsterdamAmsterdam, Netherlands

**Keywords:** membrane potential, depolarization, membrane permeability, antimicrobial, voltage-sensitive dye, potentiometric dye

## Abstract

The bacterial cytoplasmic membrane is a major inhibitory target for antimicrobial compounds. Commonly, although not exclusively, these compounds unfold their antimicrobial activity by disrupting the essential barrier function of the cell membrane. As a consequence, membrane permeability assays are central for mode of action studies analysing membrane-targeting antimicrobial compounds. The most frequently used *in vivo* methods detect changes in membrane permeability by following internalization of normally membrane impermeable and relatively large fluorescent dyes. Unfortunately, these assays are not sensitive to changes in membrane ion permeability which are sufficient to inhibit and kill bacteria by membrane depolarization. In this manuscript, we provide experimental advice how membrane potential, and its changes triggered by membrane-targeting antimicrobials can be accurately assessed *in vivo*. Optimized protocols are provided for both qualitative and quantitative kinetic measurements of membrane potential. At last, single cell analyses using voltage-sensitive dyes in combination with fluorescence microscopy are introduced and discussed.

## Introduction

Due to its central cellular role and the relatively easy accessibility for extracellular agents, the bacterial cytoplasmic membrane is a major target for antimicrobial compounds such as membrane-targeting peptides (Yeaman and Yount, 2003; Brogden, 2005; Wimley and Hristova, 2011). Disrupting the membrane function by an antimicrobial compound can simultaneously inhibit several essential processes and therefore amounts to a serious assault on a bacterial cell. This weak point is exploited by other bacteria and fungi to gain a competitive advantage in their shared habitats (Hibbing et al., 2010), and by higher organism as a part of their innate immune system (Aerts et al., 2008; Guani-Guerra et al., 2010). The membrane targeting natural peptides produced for these purposes, and synthetic ones with comparable mechanism of action, represent a largely untapped reservoir of potentially promising antibacterial lead-compounds (Giuliani et al., 2007; Riedl et al., 2011).

In many cases, dissipation of the membrane potential is either the sole mechanism of action, or contributes to the potency of the compound. This can either be caused by formation of ion-conducting membrane pores, by otherwise increasing membrane ion-permeability, or by acting as an ion carrier (Yeaman and Yount, 2003; Brogden, 2005; Wimley and Hristova, 2011). An assessment of membrane permeability is therefore one of the critical experiments in analysing the mode of action of membrane-targeting compounds. Currently, changes in membrane permeability are most commonly detected by membrane impermeable fluorescent dyes such as propidium iodide (PI) and Sytox Green. These dyes stain the cell nucleoid when the membrane integrity is severely compromised or large pores are formed (Roth et al., 1997; Sochacki et al., 2011; Stiefel et al., 2015), however, they do not detect changes in ion permeability which can nevertheless inhibit and kill bacterial cells by dissipating transmembrane potential (Wenzel et al., 2012). Changes in membrane potential can be detected by following voltage-dependent distribution of either radioactively labeled tetraphenylphosphonium ion (TPP^+^) or fluorescent dyes. Guidance for the use of TPP^+^ will be provided elsewhere by Wilmes and Sahl (Unpublished). Therefore, this method is not discussed here. Recently, membrane potential-dependent protein localization has emerged as a proxy for membrane depolarization (Strahl and Hamoen, 2010). In this manuscript, we will discuss the benefits and disadvantages of both fluorescence-based approaches, and provide experimental protocols and advice on how voltage sensitive proteins and dyes can be efficiently used in mode of action studies of membrane-targeting antimicrobials.

## Materials and methods

### Strains, media, and growth conditions

Strains and conditions for gene induction are listed in Table 1. All bacterial strains were grown either in Lysogeny Broth (LB) [10 g tryptone, 5 g/l yeast extract, 10 g/l NaCl] or Schaeffer's sporulation medium (SSM) [2.0 g/l (NH_4_)_2_SO_4_, 14.0 g/l K_2_HPO_4_, 6.0 g/l KH_2_PO_4_, 1.0 g/l Na_3_-citrate · 2H_2_0, 6.4 g/l MgSO_4_ · 7H_2_0, 1.1 mg/l (NH_4_)_5_Fe(C_6_H_4_O_7_)_2_ (ferric ammonium citrate), 0.2 g/l Casamino acids, 5 g/l D-Glucose, 0.2 g/l L-Tryptophan] at 30 or 37°C under vigorous shaking with the following exceptions. For dissipation of membrane potential with K^+^ carrier valinomycin, cells were grown in a medium composed of 10 g/l tryptone, 5 g/l yeast extract, 50 mM Hepes pH 7.5, and varying concentrations of KCl and NaCl. The time-lapse microscopy experiments were carried out in chemically defined medium (CDM) [62 mM K_2_HPO_4_, 44 mM KH_2_PO_4_, 15 mM (NH_4_)_2_SO_4_, 6.5 mM Na_3_-citrate, 0.8 mM MgSO_4_,0.6 mM MgCl_2_, 210 μM CaCl_2_, 22.5 μM MnCI_2_, 1.5 μM FeCl_3_, 0.3 μM ZnCl_2_, 0.6 μM Thiamin-HCl, 2.2 mM D-Glucose, 2.1 mM L-Glutamic acid, 6 μM L-Tryptophan; (De Jong et al., 2011)].

**Table 1:** **Strains and Plasmids**.

**Strain**	**Genotype**	**Induction**	**Source**
*B. subtilis* 168	*trpC2*	–	Barbe et al., 2009
*B. subtilis* 1981	*trpC2 minD::ermC amyE::spc Pxyl-gfp-minD*	–	Marston et al., 1998
*B. subtilis* KS64	*trpC2 amyE::spc Pxyl-gfp-minD*	0.5% xyl	This work
*B. subtilis* 2020	*trpC2 amyE::spc Pxyl-gfp-ftsZ*	0.1% xyl	Stokes et al., 2005
*B. subtilis* YK405	*trpC2 amyE::spc Pxyl.gfp-mreB*	0.8% xyl	Kawai et al., 2009
*S. aureaus* RN4220	–	–	Kreiswirth et al., 1983

### Fluorescence microscopy

For regular fluorescence microscopy, cells were grown to exponential growth phase followed by immobilization on Teflon-coated multi-spot microscope slides (Hendley-Essex) covered with a thin layer of agarose. For this aim, a solution of 1.2% electrophoresis-grade agarose was prepared in deionized H_2_O and cooled down to ~50°C. Five hundred microliters of the agarose solution was spread on a Teflon-coated slide and quickly covered with another clean, uncoated slide. The agarose was allowed to solidify for 10 min at room temperature without applied pressure. The weight of the slide, combined with the viscosity of the solution at 50°C resulted in agarose slides with a suitable thickness. Slides prepared in this manner were either used immediately, or stored maximally for 3 h in a cold humid environment in order to prevent extensive drying. Immediately before use, the slide was quickly warmed to room temperature and the uncoated slide removed by sliding it across the polymerized agarose surface. Polymerized agarose adheres well to the Teflon-coating allowing the upper clean slide to be removed with ease. 0.5 μl of a cell culture was applied to the exposed agarose surface, very briefly air-dried until the liquid-drop had evaporated, and covered with a microscopy coverslip. The applied small medium drop provides sufficient nutrients to keep the cells energized, but access to O_2_ under the coverslip was found to be limited resulting in a gradual loss of membrane potential. For these reasons, microscopy was carried out within a time window of 10 min after addition of the coverslip. The applied medium drop also provides osmolytes and we found that a combined contribution of residual non-polymerized agarose, the buffer slightly concentrated by evaporation, and the added osmolality provided by the cell suspension triggers plasmolysis-related artifacts especially in de-energized cells. The use of agarose dissolved in buffer or medium is therefore not recommended. Poly-L-lysine-coated microscopy slides were found to trigger partial dissipation of membrane potential (Strahl and Hamoen, 2010). We therefore strongly advise against the use of poly-L-lysine as a cell adhesive. Visualization of bacterial membranes was achieved by 5 min incubation in the presence of 1μg/ml of a hydrophobic membrane dye nile red (Sigma-Aldrich) prior to microscopy.

The fluorescence microscopy described in this article was carried out using Nikon Eclipse Ti (Nikon Plan Fluor 100x/1.30 Oil Ph3 DLL and Plan Apo 100x/1.40 Oil Ph3 objectives), and Applied Precision DeltaVision RT (Zeiss Plan-Neofluar 63x/1.30 Oil Ph3 and Plan-Neofluar 100x/1.30 Oil Ph3) microscopes. The images were acquired with Metamorph 6 (Molecular Devices), softWoRx Suite (Applied Precision), and analyzed using ImageJ v.1.48 (National Institutes of Health).

### Microscopic analysis of membrane potential

For the microscopic determination of membrane potential using voltage-sensitive dye 3,3′-Dipropylthiadicarbocyanine iodide [DiSC_3_(5)] (Anaspec or Sigma-Aldrich), early-mid logarithmic growth phase cell suspensions were incubated with 2 μM DiSC_3_(5) directly in the growth medium. The incubation was carried out under shaking for 5 min, immediately followed by microscopy. A final concentration of 0.5–1% DiSC_3_(5)-solvent dimethyl sulfoxide (DMSO) was found to be crucial in order to maintain appropriate solubility; lower solvent concentrations resulted in a strongly reduced cellular fluorescence. The incubation was carried out at growth temperature, and under vigorous shaking in order to maintain good energization of the cells. This step was routinely carried out with 2 ml round bottom Eppendorf tubes containing 200 μl cell suspension in a thermomixer. To provide sufficient aeration, the lids of the tubes were perforated. When dissipation of membrane potential was tested, a compound of interest was added in parallel to DiSC_3_(5). Addition of 5 μM gramicidin (a mixture of gramicidin A, B, C, and D) was routinely used as a positive control. This peptide mixture triggers a rapid and full dissipation of membrane potential by forming small cation specific channels (Kelkar and Chattopadhyay, 2007). If longer incubation times are required, the cell suspension can be pre-treated with the compound of interest, followed by 5 min staining with DiSC_3_(5). The imaging of DiSC_3_(5)-stained cells was carried out using commonly available Cy5-filter sets.

In contrast to another voltage sensitive dye DiBAC_4_(3) which will be discussed later, DiSC_3_(5) does not exhibit strong affinity to glass surfaces and regular uncoated microscopy coverslips can be used. Strong affinity toward PDMS, which is commonly used to fabricate microfluidic devises, has however been observed by us and others (Prindle et al., 2015) thus complicating the use of this dye in a microfluidic setup.

### Fluorescence quantification from micrographs

The quantification of cellular DiSC_3_(5) fluorescence was carried out in a semi-automated manner using ImageJ. The fluorescent images were first background-subtracted in order to remove signal originating from unincorporated dye and medium. The phase contrast images acquired in parallel to fluorescence images were used to identify cells as regions of interest (ROI), for which DiSC_3_(5) fluorescence was measured. For micrographs in which cells were well separated, an automated cell detection, carried out as described in the ImageJ-manual in the context of image thresholding and particle detection, was found to be sufficient. In most cases, however, cells adhered to each other or grew in chains. In this case the ROI's were determined manually for each cell in an image field by drawing a line with an appropriate pixel width along the length axis of the cells. When applying this method, care was taken to ensure that the ROI maximally covered the cell while still remaining entirely within boundaries of the cell. At last, the average fluorescence intensity of each cell (ROI) was measured using the fluorescence images. All the steps described above were carried out with standard ImageJ but an ImageJ-plugin ObjectJ can be used to further automate this process (Vischer et al., 2015).

### Time-lapse microscopy

The time-lapse microscopy was carried out as described in De Jong et al. (2011) with the following modifications. Instead of growing *B. subtilis* pre-culture in TLM-medium, cells were grown overnight in SMM-medium, followed by 1:10 dilution in CDM-medium. The cultures adapted to growth in CDM were used to inoculate the time-lapse microscopy slides in an appropriate dilution. The principal methodology regarding the preparation of time lapse-microscopy slides is described in De Jong et al. (2011). For the time-lapse microscopy using a voltage-dependent dye, 10 μM Bis-(1,3-dibutylbarbituric acid) trimethine oxonol [DiBAC_4_(3)] (Anaspec or Sigma-Aldrich) was included in the solid time-lapse growth medium. This dye was dissolved in dimethylformamide (DMF) and a final solvent concentration of 1% DMF was maintained in the growth medium. DiBAC_4_(3) exhibits considerable affinity to glass surfaces which strongly interferes with fluorescent imaging. This binding was supressed by pre-treating the coverslips accordingly (see below). Commonly available FITC or GFP filters were used to image DiBAC_3_(4)-stained cells.

### Polydopamine coating

To prevent binding of DiBAC_4_(3) to glass surfaces, the coverslips used in combination with DiBAC_4_(3) were coated with polymerized L-dopamine (Zhang et al., 2013; Strahl et al., 2015). In brief, a large drop of a fresh solution of 2 mg/ml L-dopamine in 1 mM Tris pH 8.0 was spread on the required coverslip surface, followed by an incubation for 30 min at room temperature. Subsequently, non-polymerized L-dopamine and Tris was removed by aspiration and submersion of the coverslip in deionized H_2_O. Remaining H_2_O was removed by evaporation at 37°C for 30 min. Coverslips pre-treated in this manner were stored in a dark, dry, and dust-free environment.

### Fluorometric measurement of membrane potential using DiSC_3_(5)

The fluorometric measurements of membrane potential using voltage-sensitive dye DiSC_3_(5) were carried out on black polystyrene microtiter plates (Labsystems) using BMG Fluostar Optima fluorometer equipped with 610 ± 5 nm excitation, and 660 ± 5 nm emission filters. However, any temperature-regulated fluorometer capable for excitation and detection at the required wavelengths should in principle be compatible. The measurements were routinely carried out directly in LB medium supplemented with 0.5 mg/ml BSA. Addition of BSA was found to reduce absorption of DiSC_3_(5) to polystyrene surfaces. An optimal cell density and dye concentration were found to be the key parameters determining the signal-to-noise ratio in this assay. For *B. subtilis* an OD_600_ of 0.2, combined with 1 μM DiSC_3_(5) was found to provide a good fluorescence intensity difference between fully energized and depolarized cells in LB. For *S. aureus* an OD_600_ of 0.3 is recommended. When adapting this assay to new bacterial species or media, the optimal cell density and dye concentration should first be determined.

To carry out the assay, cells were grown in LB medium until logarithmic growth was obtained, and diluted to an OD_600_ of 0.2 in a pre-warmed LB supplemented with 0.5 mg/ml BSA. One hundred and thirty five microliters the diluted cells were transferred to the microtiter plate and the fluorescence was followed for 2–3 min in order to obtain values for medium and cell background fluorescence. After obtaining a baseline, DiSC_3_(5) dissolved in DMSO was added to each well to a final concentration of 1 μM DiSC_3_(5) and 1% DMSO. Maintaining 1% DMSO was critical for good solubility and fluorescence of the dye. The fluorescence quenching was measured until a stable signal intensity was achieved, followed by addition of the compound of interest. One micro molar of channel-forming peptide gramicidin was found to be a suitable positive control. If a rapid depolarization was observed, no compensation for cell growth was required. If the depolarization was slow, cell growth was found to result in a gradual shift in the fluorescence values observed with the untreated control sample. An inhibition of cell growth with 5 μg/ml chloramphenicol was found to suppress this shift and resulted in stable fluorescence levels. Maintaining appropriate aeration during the measurement was found to be crucial to maintain stable fluorescence levels for polarized cells. For these reasons, the microtiter plates were subjected to vigorous shaking between each measurement point. Following this regime, the development of membrane potential upon addition of a compound of interest can be followed for up to 60 min.

### Calibration of DiSC_3_(5) assay

For the calibration of DiSC_3_(5) assay, Nernst equation (see below) was used to calculate medium K^+^ concentrations (${\text{K}}_{\text{out}}^{+}$) which correspond to the desired K^+^ equilibrium potentials. Three hundred micro molars was used as a close approximation for the cellular K^+^ concentration (${\text{K}}_{\text{in}}^{+}$) of *B. subtilis* (Whatmore et al., 1990).

$$\begin{array}{l} V_{Eq.}\;=\;\frac{RT}{zF}\text{ln}\left(\frac{{\left[K^{+}\right]}_{out}}{{\left[K^{+}\right]}_{in}}\right) \end{array}$$

The calculated values (Table 2) were used to prepare media with different K^+^ concentrations ($K_{\text{out}}^{+}$). To maintain an identical ionic strength in all media (300 mM), NaCl was added accordingly. K^+^ present in yeast extract (~6 mM for final concentration for 5 g/l) was taken into calculation. *B. subtilis* was grown in the different media to a logarithmic growth phase, diluted to an OD_600_of 0.2, and subjected to the fluorometric DiSC_3_(5) assay as descried above. The membrane potential was dissipated by addition of 5 μM K^+^ carrier valinomycin. This concentration was found to be sufficient to fully depolarize *B. subtilis* cells without interfering significantly with DiSC_3_(5) fluorescence. Upon addition, the increased membrane K^+^ permeability results in dissipation of the membrane potential to values pre-determined by the K^+^ gradient across the membrane. The resulting calibration ladder was used to convert relative DiSC_3_(5) fluorescence values into absolute values.

**Table 2:** **Calculated K^**+**^ equilibrium potentials used in calibration of DiSC_**3**_(5) assay**.

*K^+^*_in_(mM)	300	300	300	300
*K^+^*_out_(mM)	300	46	18.1	7.1
*V*_*Eq*._(mV)	0	−50	−75	−100

## Results and discussion

### Delocalization of MinD as a proxy for membrane depolarization

**Figure 1:**
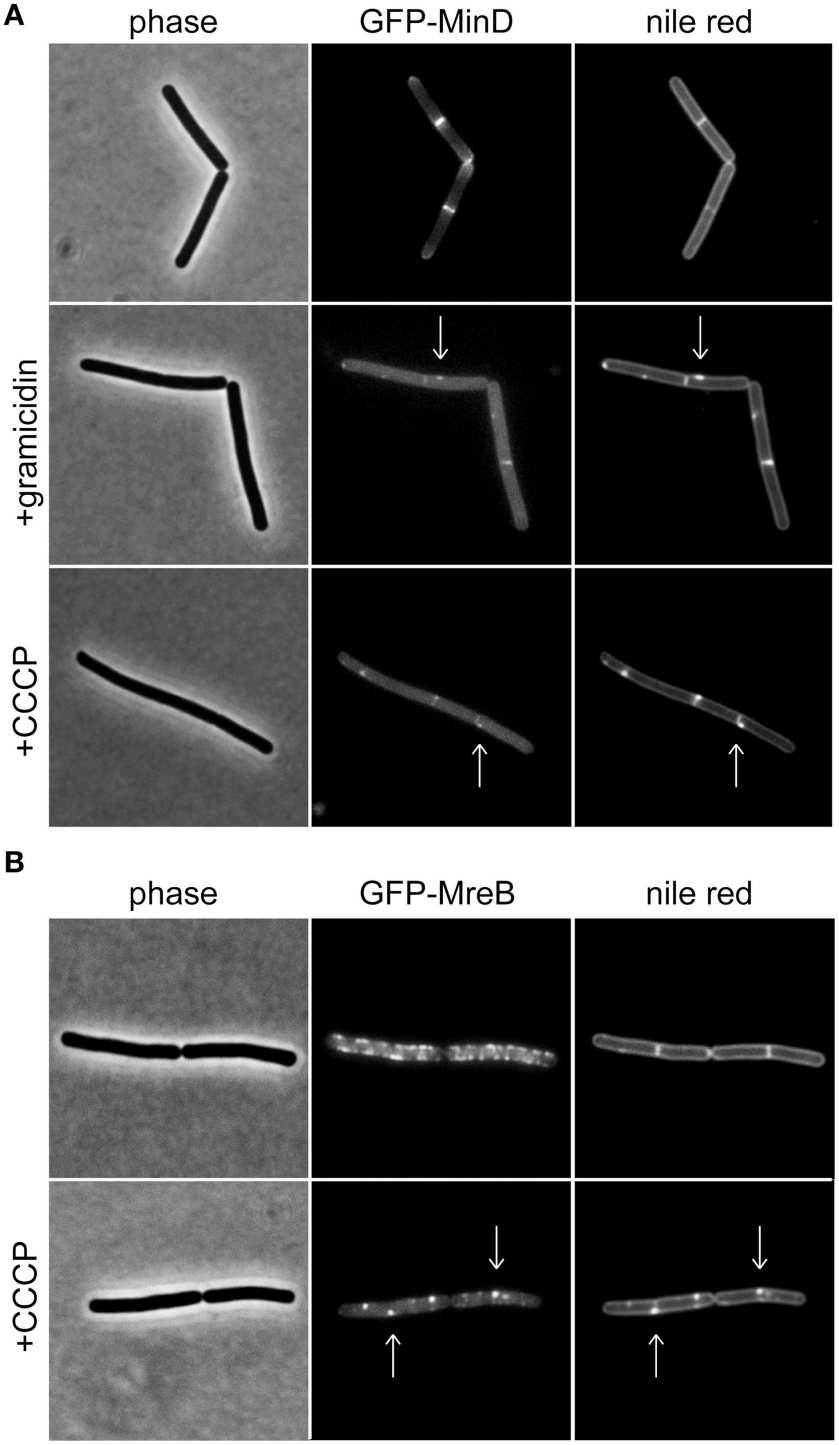
**Membrane potential-dependent localization pattern of MinD and MreB. (A)** Phase contrast images (left panel) of *B. subtilis* cells expressing GFP-MinD (middle panel), and stained with fluorescent membrane dye nile red (right panel) in the absence and presence of the small cation channel forming antimicrobial peptide gramicidin (1 μM), or H^+^ ionophore CCCP (100 μM). Note the loss of polar membrane associated localization pattern upon depolarization, and a few remaining foci which colocalize with fluorescent nile red foci (arrows). **(B)** Phase contrast images (left panel) of *B. subtilis* cells expressing GFP-MreB (middle panel), and stained with fluorescent membrane dye nile red (right panel) in the absence and presence of CCCP (100 μM). Note the clustering of the protein upon depolarization which triggers the fluorescent nile red foci (arrows). Strains used: *B. subtilis* KS64 (GFP-MinD), and *B. subtilis* YK405 (GFP-MreB).

Previously, we have shown that the membrane potential is crucial for correct cellular localization of certain membrane proteins in *B. subtilis* and *E. coli* (Strahl and Hamoen, 2010). One of the identified proteins was the cell division regulator MinD (Marston et al., 1998; Rothfield et al., 2005). Under normal growth conditions, a *B. subtilis* GFP-MinD fusion protein is membrane-associated, and enriched at cell poles and cell division sites. Upon depolarization, the membrane association of this protein is destabilized resulting in a clear loss of polar localization pattern and an increasingly cytoplasmic GFP signal (Strahl and Hamoen, 2010; Figure 1A). Based on this discovery, fluorescence microscopy using MinD localization as a proxy for changes in membrane potential has emerged as a convenient tool (Chimerel et al., 2012; Eun et al., 2012; Wenzel et al., 2012, 2013; Foss et al., 2013). While localization of MinD is a useful indicator for membrane depolarization, there are two complications, which should be taken into consideration when using this method.

MinD is an ATP-binding protein and both binding and hydrolysis of ATP alters its localization pattern (Karoui and Errington, 2001; Strahl and Hamoen, 2010). As a consequence, reduced cellular ATP levels could be mis-interpreted as membrane depolarization. As shown in Figure 1A, dissipation of membrane potential results in an increasingly cytoplasmic GFP-MinD fluorescence signal but a few remaining membrane associated fluorescent foci are also observed. *B. subtilis* actin homolog MreB, a protein involved in cell wall synthesis, is also delocalized upon membrane depolarization (Strahl and Hamoen, 2010). Recently, we have shown that delocalization of MreB causes a pattern of lipid domains to emerge which are characterized by an increased membrane fluidity, and therefore attract membrane dyes and proteins such as MinD (Figure 1B; Strahl et al., 2014). Thus, the change in localization of MinD upon membrane depolarization is caused by a combination of two factors: a direct interference with MinD-membrane binding (resulting in cytoplasmic signal), and an indirect effect caused by delocalization of MreB (resulting in localization in membrane foci). While both patterns are caused by membrane depolarization, care should be taken when interpreting an observed delocalization into foci as an indicator for membrane depolarization. In this case, a mere delocalization of MreB rather than dissipation of membrane potential is an alternative explanation.

For these reasons, we recommend that localization analysis of MinD should be used in combination with more direct techniques such as those introduced in the following sections. MinD-localization assay does, however, provide a rapid method to rule out potential membrane depolarization effects, and is a valuable complementary tool to augment the dye-based techniques especially when extensive interference with dye-fluorescence is observed (discussed below).

### Membrane potential measurement using voltage sensitive dye DiSC_3_(5)

**Figure 2:**
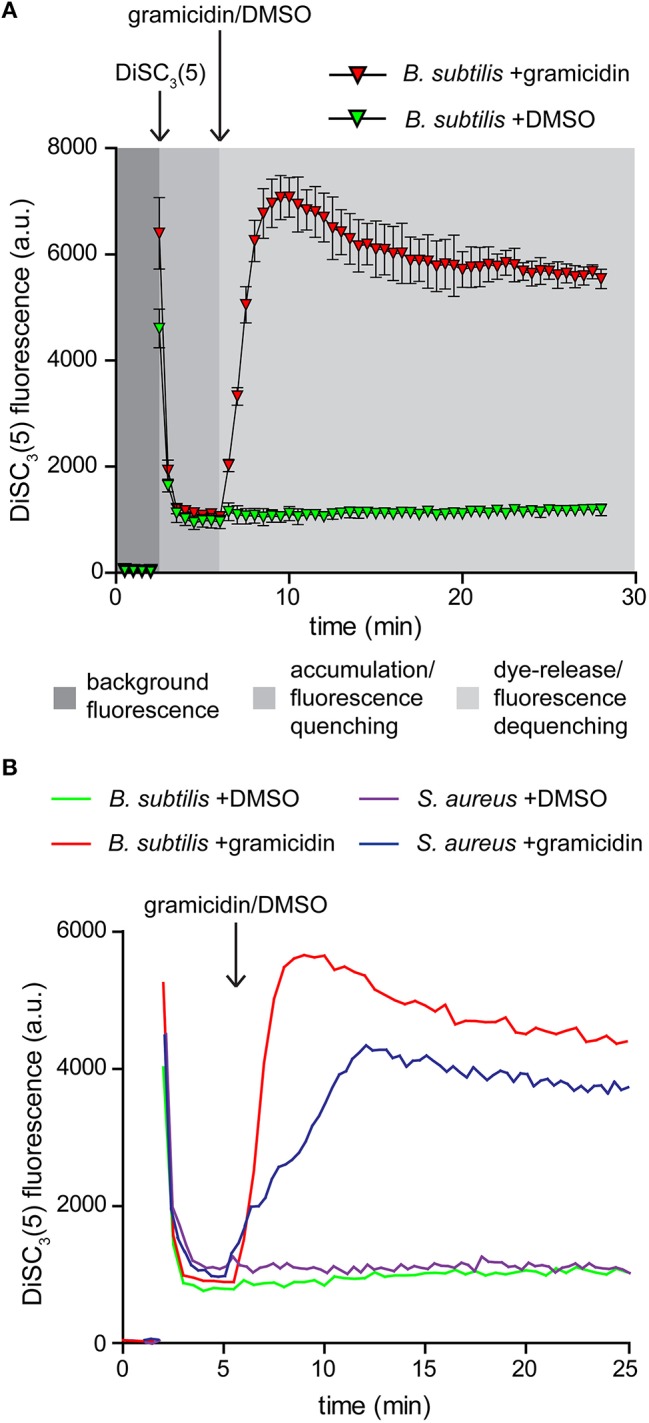
**Membrane potential sensitive fluorescent dye DiSC_**3**_(5). (A)** Fluorescence intensity changes of DiSC_3_(5) in a cell suspension. The time points of dye and gramicidin addition are highlighted with arrows. Note the strong quenching of DiSC_3_(5) fluorescence upon accumulation in cells, and the dequenching upon membrane depolarization. The solvent used to solubilize gramicidin (DMSO) has no influence on the fluorescence intensity levels. The graph depicts average and standard deviation of three technical replicates. **(B)** A comparison of fluorescence intensity changes of DiSC_3_(5) upon accumulation in polarized *B. subtilis* and *S. aureus* cells, and upon depolarization with gramicidin (1 μM). The time point of gramicidin addition is highlighted with an arrow. Strains used: *B. subtilis* 168 (wild type) and *S. aureus* RN4220 (wild type).

DiSC_3_(5) is a cationic membrane-permeable fluorescent dye. The charged nature combined with sufficient hydrophobicity to penetrate lipid bilayers allows this dye to act as a potentiometric probe and accumulate in polarized cells until a Nernstian equilibrium is achieved (Waggoner, 1976, 1979; Bashford, 1981; Ehrenberg et al., 1988). The strong accumulation in energized cells results in quenching of the overall fluorescence of the cell suspension. Upon depolarization, the dye is rapidly released into the medium resulting in dequenching that can be followed fluorometrically (Figure 2A; Singh and Nicholls, 1985; Shapiro, 1994).

Two critical parameters should be considered when using this dye for analysing membrane depolarization triggered by an antimicrobial compound. To obtain a strong response based on fluorescence quenching, both the cell density and the dye concentration should be optimized. Higher dye concentration results in stronger overall fluorescence signals but the fluorescence difference between polarized and depolarized cells is reduced. The same is observed for an increased cell density. We found a concentration of 1 μM DiSC_3_(5), combined with an OD_600_ of 0.2 to be optimal for *B. subtilis*. For *S. aureus* a slightly elevated cell density of OD_600_ = 0.3 provided comparable signals (Figure 2B).

**Figure 3:**
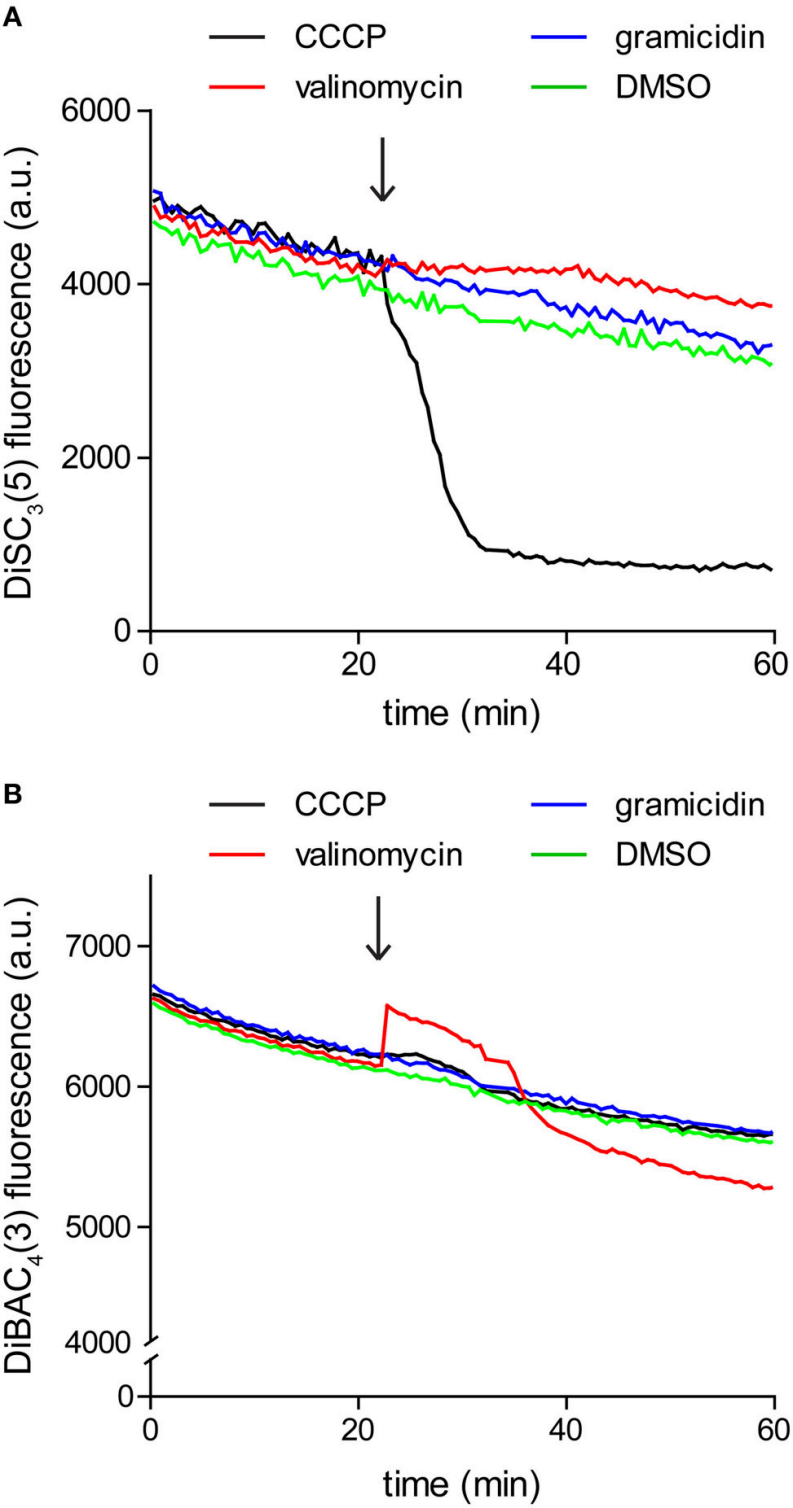
**Interference between dye fluorescence and ionophores. (A)** Fluorescence intensity of 1 μM DiSC_3_(5) in LB medium upon addition of channel-forming peptide gramicidin (1 μM), K^+^ carrier depsipeptide valinomycin (5 μM), and a non-peptide protonophore CCCP (30 μM). The time point of addition is highlighted with an arrow. Note the gradual decay of fluorescence signal due to binding on the polystyrene-surface of the microtiter plate, and the strong reduction of fluorescence upon addition of CCCP, which disqualifies the use of CCCP in combination with DiSC_3_(5). **(B)** Fluorescence intensity of 10 μM DiBAC_4_(3) in LB medium upon addition of channel forming peptide gramicidin (1 μM), K^+^ carrier depsipeptide valinomycin (5 μM), and the non-peptide protonophore CCCP (30 μM). In case of this dye, interference is observed with valinomycin.

Since the assay is based on fluorescence quenching, potential interaction between the dye and the compound of interest, or strong light absorption could influence the measured fluorescence. For these reasons it is crucial to verify that the compound of interest does not significantly alter the dye fluorescence at the used concentrations. We found it easiest to perform this control by simply repeating the fluorometric assay in the absence of cells. As shown in Figure 3, gramicidin and K^+^-specific carrier valinomycin are compatible with DiSC_3_(5) assay whereas the commonly used protonophore CCCP is incompatible. In contrast, Valinomycin cannot be used in combination with another frequently used voltage-sensitive dye DiBAC_3_(4) (discussed later).

### Calibration of the fluorometric DiSC_3_(5) assay

**Figure 4:**
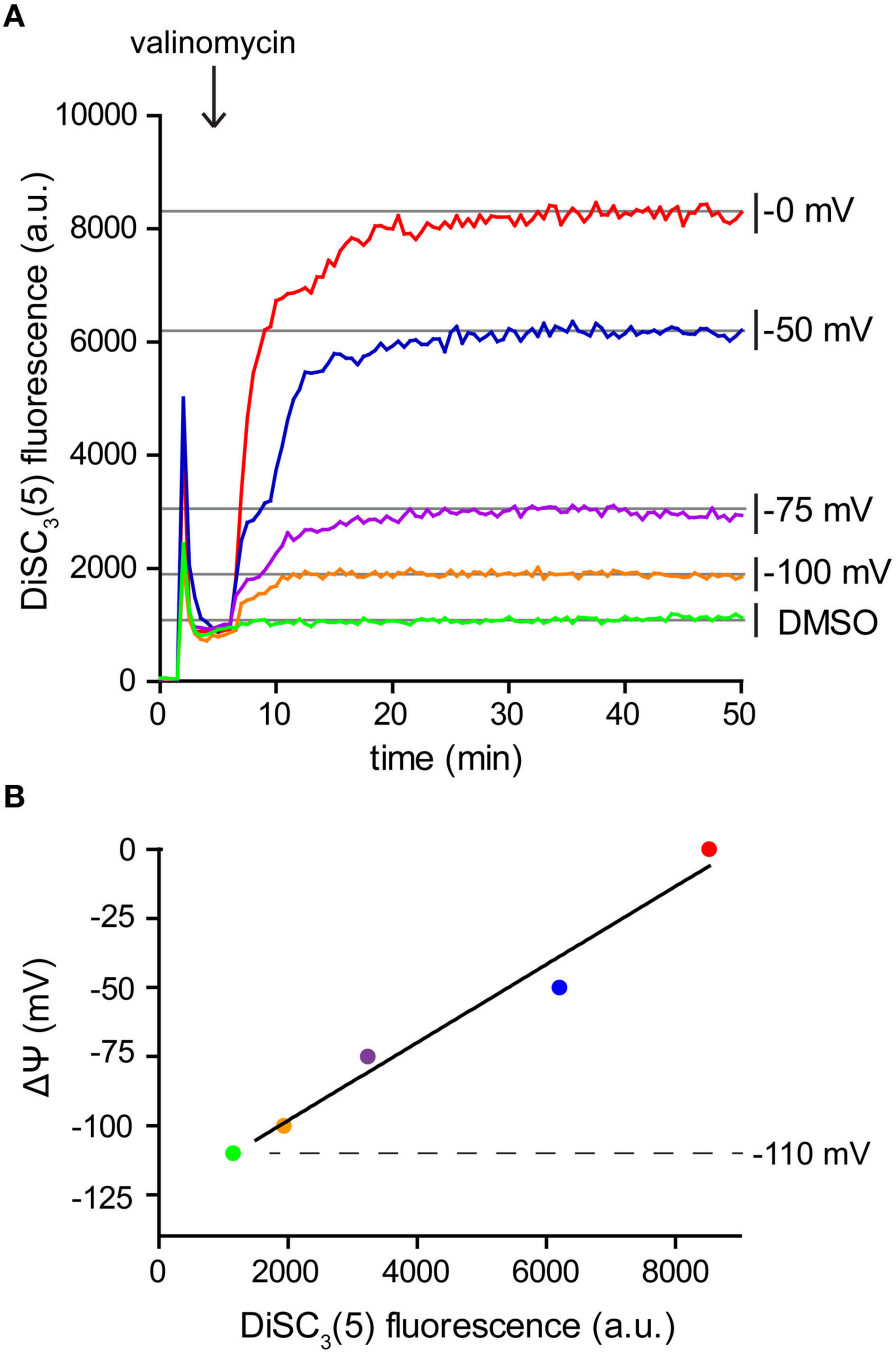
**Calibration of DiSC_**3**_(5) assay. (A)** Fluorescence intensity of DiSC_3_(5) in *B. subtilis* cell suspensions in media with varying K^+^ concentrations. Upon addition of K^+^ carrier valinomycin (4 μM), membrane potential reaches stable levels pre-determined by the K^+^ gradient across the membrane (see Table 2). The time point of valinomycin addition is highlighted with an arrow. **(B)** The fluorescence levels obtained from the experiment shown in panel **(A)** can be used to calibrate the arbitrary DiSC_3_(5) fluorescence values. The estimated membrane potential for untreated *B. subtilis* cells (−110 mV) is indicated with a dashed line. Strain used: *B. subtilis* 168 (wild type).

Since DiSC_3_(5) follows a Nernstian distribution across the membrane, its fluorescence can also be used for a quantitative measurement of the membrane potential. For this aim, partial depolarization triggered by the K^+^ carrier valinomycin serves as a convenient method to generate a calibration curve. Following the Goldman-Hodgkin-Katz voltage equation, membrane potential is determined by the relative contribution of membrane gradients for each ion. The extent, to which an ion gradient contributes to the overall membrane potential, depends on the permeability (both passive and active) of the membrane to the individual ion species. Under normal conditions, the transport of H^+^ across the membrane greatly surpasses the contribution of other ions. This is mainly due to the high H^+^ transport activity of the respiratory chain. As a consequence, the membrane potential is normally dominated by the H^+^ gradient (Mitchell, 1961; Saraste, 1999). This changes upon addition of the K^+^-specific carrier valinomycin (Shapiro, 1994). When supplied at sufficient concentrations, the relative permeability of the cell membrane for K^+^ is increased to an extent in which the K^+^ gradient across the membrane becomes the predominant factor. Upon addition of valinomycin, the membrane potential now equilibrates with the K^+^ gradient resulting in a level which can be calculated using the Nernst-equation (see Table 2). K^+^ gradient as such can easily be modified by altering the medium K^+^/Na^+^-ratio since cells strive to maintain a stable cytoplasmic K^+^ concentration in media with comparable osmolality (Whatmore et al., 1990). This method provides the means to measure DiSC_3_(5) fluorescence at different membrane potential levels (Figure 4A), and thus to calibrate the DiSC_3_(5) assay (Figure 4B; Singh and Nicholls, 1985; Vecer et al., 1997; Breeuwer and Abee, 2004). Using this approach, we estimated the membrane potential of *B. subtilis* to reach ~ −110 mV under the growth conditions used in our experiments. This value is in good agreement with previously published estimates (Hosoi et al., 1980; Zaritsky et al., 1981).

### Single cell analysis using DiSC_3_(5)

**Figure 5:**
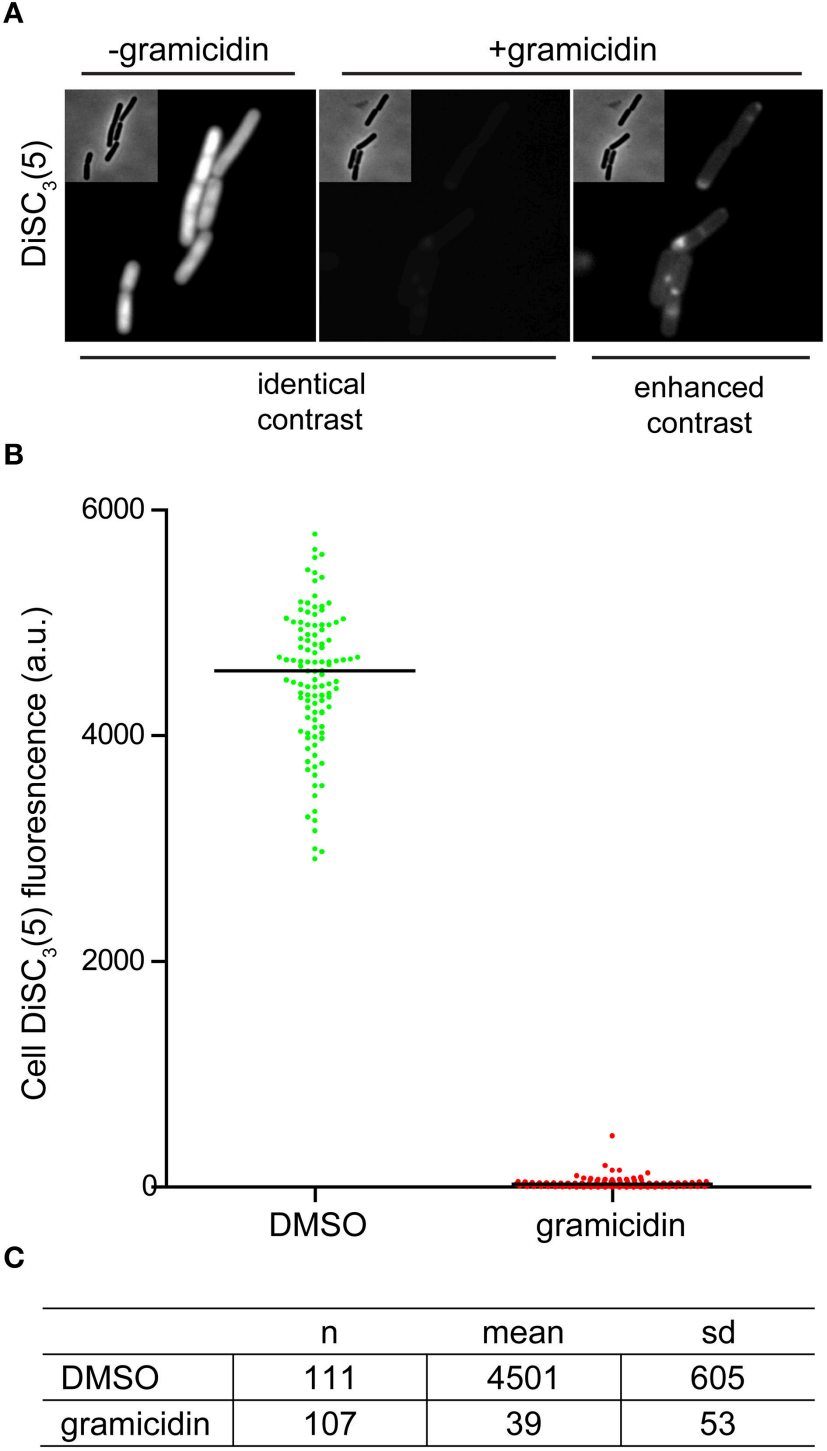
**Microscopic single-cell measurement of membrane potential using DiSC_**3**_(5). (A)** Fluorescence microscopy of DiSC_3_(5)-stained *B. subtilis* cells in the absence (left panel) and presence (right panels) of the small cation channel forming antimicrobial peptide gramicidin (5 μM). In the left, the polarized and depolarized cells are depicted with identical contrast settings providing a direct comparison. On the right, the contrast of the fluorescence image is enhanced in order to visualize the remaining staining. **(B)** The cellular DiSC_3_(5) fluorescence was quantified for logarithmic growth phase cells in the presence and absence of gramicidin (5 μM). The column scatter plot depicts the individual single-cell intensities and the median thereof. **(C)** The mean and standard deviation of the DiSC_3_(5)-fluorescence for the individual cells analyzed in graph **(B)** are shown. Strain used: *B. subtilis* 168 (wild type).

As mentioned earlier, changes in localization of GFP-MinD provides a convenient assay to test candidate compounds for their ability to dissipate the membrane potential. However, delocalization is a relatively subjective measure. As an alternative, direct fluorescence microscopy of DiSC_3_(5)-stained cells provides a comparably simple and rapid assay (Figure 5A). The spectral properties of DiSC_3_(5) allows fluorescence microscopy to be performed with commonly available filters used to detect Cy5 and similar fluorophores. Although accumulation of DiSC_3_(5) does cause self-quenching, the remaining cellular fluorescence is still very strong and provides a good signal to background ratio. Upon depolarization, the change in cellular fluorescence is large (>100-fold) thus providing an unambiguous readout (Figures 5B,C).

**Figure 6:**
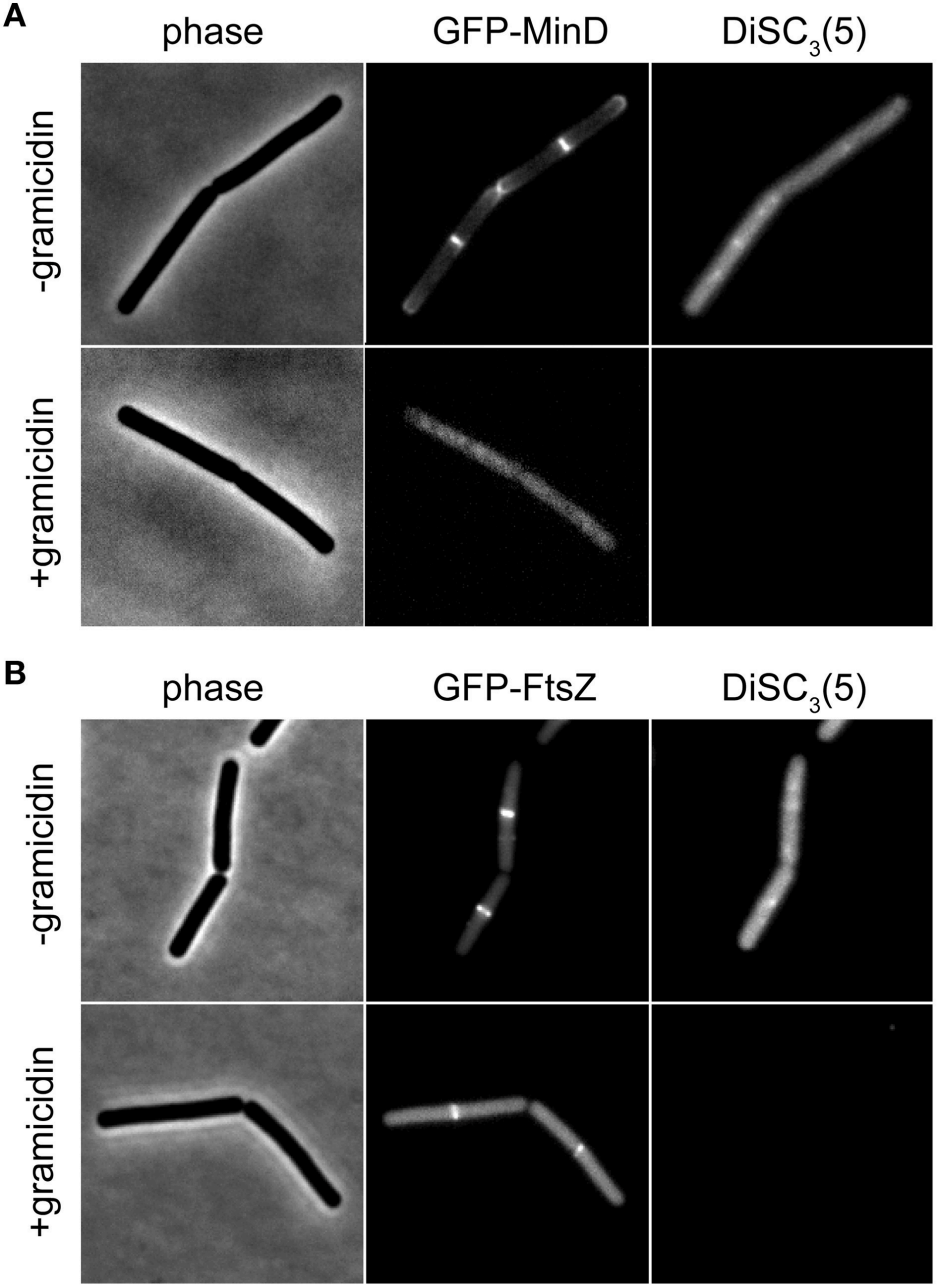
**Compatibility of DiSC_**3**_(5) and GFP in microscopic single-cell experiments. (A)** Phase contrast image (left panel) of cells expressing GFP-MinD (middle panel) and stained with DiSC_3_(5) (right panel) in the absence and presence of gramicidin (5 μM). Note the strong decrease in DiSC_3_(5) fluorescence and delocalization of MinD upon depolarization. **(B)** Phase contrast image (left panel) of cells expressing GFP-FtsZ (middle panel) and stained with DiSC_3_(5) (right panel) in the absence and presence of gramicidin (5 μM). Note the reduced septal signal of FtsZ upon depolarization. Strains used: *B. subtilis* KS64 (GFP-MinD), and *B. subtilis* 2020 (GFP-FtsZ).

The speed and extent of membrane depolarization by an antimicrobial compound is frequently used in discussing pore formation as a potential mechanism of action (Silverman et al., 2003; Spindler et al., 2011). Since normal fluorometric assays only measure the cell population average, a cell-to-cell heterogeneity can result in seemingly slow depolarization kinetics even when full and rapid depolarization of individual cells takes place. By detecting such heterogeneity, the single cell analysis using DiSC_3_(5) provides a valuable control experiment to support conclusions drawn from fluorometric measurements. At last, the far-red fluorescence of DiSC_3_(5) is compatible with simultaneous detection of GFP. Changes in membrane potential upon antimicrobial challenge, and the consequences on cellular organization and protein localization can therefore be directly correlated on a single-cell level (Figure 6).

### Time-lapse microscopy using voltage-sensitive dyes

Time-lapse microscopy, in combination with microfluidic devices, is developing into a powerful tool in antibiotic mode of action studies. The strength of this approach is the direct and continuous observation of individual cells upon antimicrobial challenge (Sochacki et al., 2011; Barns and Weisshaar, 2013; Nonejuie et al., 2013). Very recently, time-lapse microscopy was used in combination with a cationic dye thioflavin T to analyse fluctuation of membrane potential in bacterial cell communities (Prindle et al., 2015). The voltage-dependent behavior of this dye is, however, at this stage only poorly characterized.

Several dyes commonly used to stain bacterial cells are growth inhibitory, and can therefore not be combined with time-lapse microscopy. This also turned out to be true for DiSC_3_(5) and a clear growth inhibition of *B. subtilis* was observed which makes DiSC_3_(5) incompatible with time-lapse experiments. However, another applicable voltage-dependent dye [DiBAC_3_(4)] does not inhibit growth at concentrations up to 10 μM (data not shown).

**Figure 7:**
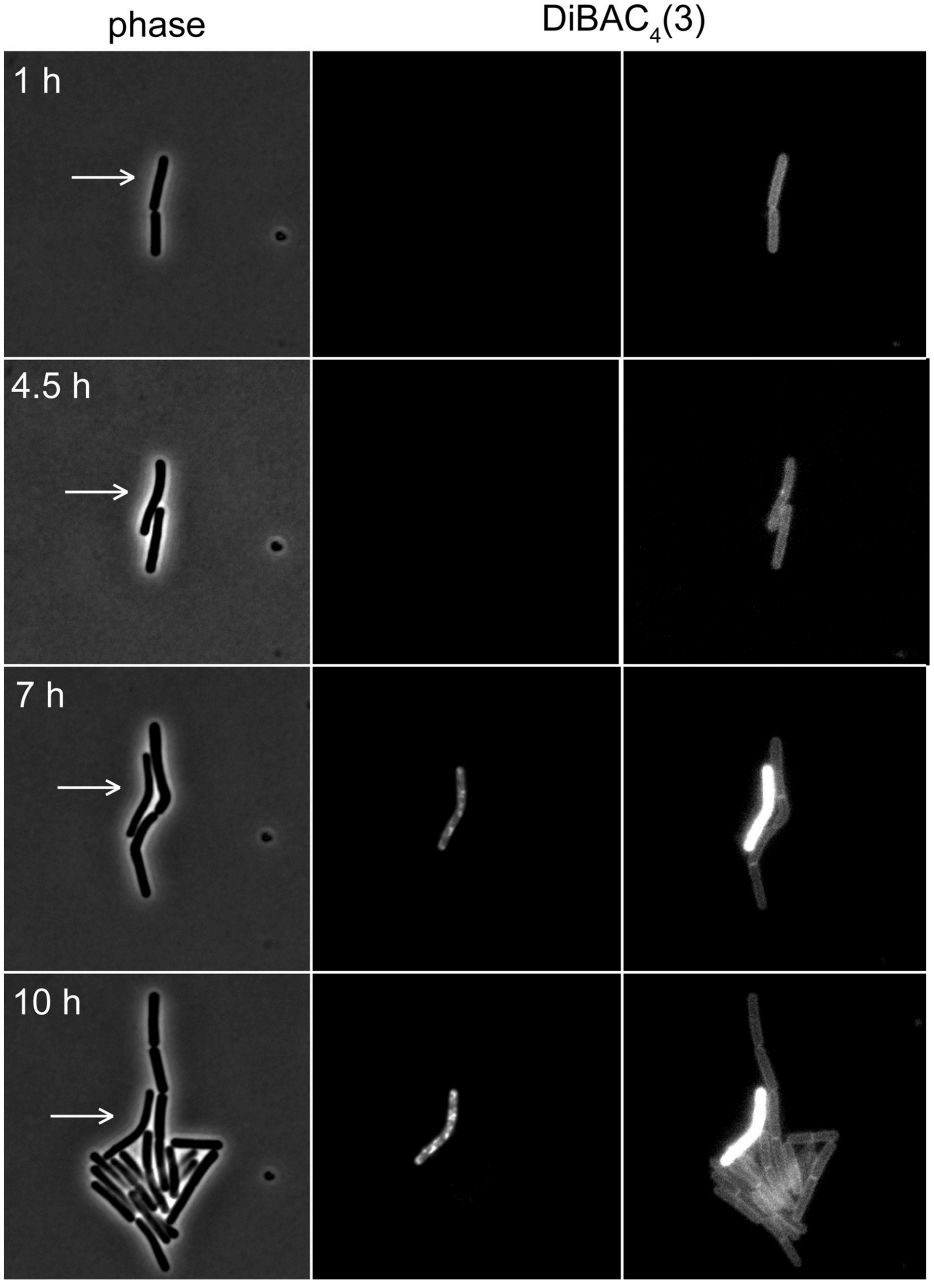
**Time-lapse microscopy using voltage sensitive dye DiBAC_**4**_(3)**. Selected images of a time-lapse microscopy experiment carried out with *B. subtilis* cells stained with DiBAC_4_(3). Phase contrast images (left panel) and DiBAC_4_(3) fluorescence images (right panels) are depicted. Due to the strong fluorescence intensity difference between polarized and depolarized cells, the images are shown with two different contrast settings. High fluorescence intensity of DiBAC_4_(3) indicates low membrane potential. An individual cell that failed to enter logarithmic growth and subsequently undergoes membrane depolarization, is indicated with an arrow. See Supplementary Movie 1 for an example of a whole time-lapse series. Strain used: *B. subtilis* 168 (wild type).

The voltage-sensitivity of DiBAC_3_(4) is based on the same fundamental principle as DiSC_3_(5) (Brauner et al., 1984; Epps et al., 1994). In contrast to positively charged DiSC_3_(5), however, DiBAC_3_(4) is an anion and the response of this dye to the membrane potential is accordingly opposite. In polarized cells, the dye is excluded from the cells due to the negative charge. This results in low fluorescent staining of well-energized cells. Upon depolarization, the dye can now enter the cell and strongly stain the cellular membranes. Indeed, when DiBAC_3_(4) was included in a regular time-lapse microscopy setup, this dye could be used as a reporter for membrane potential levels even in long term experiments spanning over 17 h (see Figure 7 and Supplementary Movie 1). Thus, voltage dependent dyes can be used in combination with time-lapse experiments allowing continuous monitoring of membrane potential at a single cell level.

## Summary

In this manuscript, we describe several techniques to analyse bacterial membrane potential *in vivo*. While the applied spectroscopic and microscopic techniques are not novel, the emphasis here is on providing readily optimized generic assays for antibiotic research. The benefits and weaknesses of the individual assays, their analytical basis, and the underlying theory are discussed in order to provide a good starting point for a reader without extensive background knowledge. The methods presented here should be easy to implement and utilize commonly available equipment such as fluorescence plate reader and a regular fluorescence microscope. Every individual experiment is different and slight modifications to the presented generic protocols are likely to be necessary. We hope that the included details, controls, and the discussion will provide the means to easily adopt the introduced methods to the specific research questions. The techniques described here are optimized for *B. subtilis* but are, in principle, well transferable for use with other bacterial species.

## Author contributions

JT, DG, KS, and HS carried out the experiments and analyzed the data; HS and LH designed the project; HS wrote the paper.

## Funding

HS was supported by Wellcome Trust Institutional Strategic Support Funds (ISSF) grant 105617/Z/14/Z, DG by Biotechnology and Biological Sciences Research Council (BBSRC) DTP Studentship BB/J014516/1, and LH by Netherlands Organisation for Scientific Research (NWO) grant STW-Vici 12128.

### Conflict of interest statement

The authors declare that the research was conducted in the absence of any commercial or financial relationships that could be construed as a potential conflict of interest.

## Supplementary material

Supplementary Movie 1 **Time-lapse microscopy using voltage sensitive dye DiBAC_4_(3)**. Time-lapse microscopy of *B. subtilis* cells stained with DiBAC_4_(3) over a time period of 17 h. Depicted are phase contrast images (left panel) and DiBAC_4_(3) fluorescence images (right panel) with a time resolution of 5 min. High fluorescence intensity of DiBAC_4_(3) indicates low membrane potential levels. Note the overall reduction of membrane potential upon entry into nutrient starvation-induced stationary growth phase. Strain used: *B. subtilis* 168 (wild type).
